# On the Syntactic Monoids Associated with a Class of Synchronized Codes

**DOI:** 10.1155/2013/691439

**Published:** 2013-12-24

**Authors:** Shou-feng Wang

**Affiliations:** School of Mathematics, Yunnan Normal University, Kunming, Yunnan 650500, China

## Abstract

A complete code *C* over an alphabet *A* is called *synchronized* if there exist *x*, *y* ∈ C* such that xA*∩A*y⊆C*. In this paper we describe the syntactic monoid Syn(*C* ^+^) of *C* ^+^ for a complete synchronized code *C* over *A* such that *C* ^+^, the semigroup generated by *C*, is a single class of its syntactic congruence *P*_*C*^+^_. In particular, we prove that, for such a code *C*, either *C* = *A* or Syn(*C* ^+^) is isomorphic to a special submonoid of *𝒯* ^*l*^(*I*) × *𝒯* ^*r*^(Λ), where *𝒯* ^*l*^(*I*) and *𝒯* ^*r*^(Λ) are the full transformation semigroups on the nonempty sets *I* and Λ, respectively.

## 1. Introduction

Theory of codes is an important branch in the field of information science. Many methods, including combinatorics methods, analysis methods, and algebraic methods, are applied to study codes. As a kind of algebraic methods, it is effective to study some kinds of codes by considering syntactic monoids of the semigroups and monoids generated by these codes.

As we have known, prefix codes have fundamental importance in theory of codes. Many authors are devoted to the investigation of prefix codes by using several methods (cf. [1–4]). In particular, Petrich et al. [4] investigated maximal prefix codes by considering the syntactic monoids of the semigroups generated by them in 1996. They described the semigroup structure of the syntactic monoid Syn(*C* ^+^) of *C* ^+^, the semigroup generated by a maximal prefix code *C* for which *C* ^+^ is a single class of the syntactic congruence *P*_*C*^+^_.

On the other hand, synchronized codes are also important both in theory and in applications. Many interesting results are obtained on this class of codes in the text of Berstel et al. [1]. Recently, Liu [3] investigated synchronized codes by algebraic methods and obtained an algebraic characterization of complete synchronized codes (see Lemma 5 in this paper). Furthermore, Liu [3, 5] also studied some generalizations of synchronized codes.

In this paper, by using the algebraic characterization of complete synchronized codes obtained in Liu [3] and some techniques developed in Petrich et al. [4], we give a description of the syntactic monoid Syn(*C* ^+^) of *C* ^+^ for a complete synchronized code *C* over an alphabet *A* such that *C* ^+^ is a single class of its syntactic congruence *P*_*C*^+^_.

## 2. Preliminaries

A semigroup *S* is a *left zero semigroup* if *ab* = *a* for any *a*, *b* ∈ *S*. Dually, we have *right zero semigroups*. A *rectangular band* is a semigroup which is isomorphic to a direct product of a left zero semigroup and a right zero semigroup. For rectangular bands, we have the following obvious result.

**Lemma 1 (see [6])**

Let *S* be a rectangular band. Then *axa* = *a* for any *a*, *x* ∈ *S*. As a consequence, *aSa* = {*a*} for any *a* ∈ *S*. In particular, if *S* has an identity *e*, then *S* = {*e*}.

An *ideal* of a semigroup *S* is a nonempty subset *I* of *S* satisfying that the union of *IS* and *SI* is contained in *I*. Recall that the unique minimum ideal (with respect to set inclusion) of a semigroup *S* (if exists) is called *the kernel of S*. For the kernel of a semigroup, we have the following.

**Lemma 2 (see [6])**

If the kernel of a semigroup *S* only consists of idempotents, then this kernel is a rectangular band.

Let *S* be a semigroup. A function *λ* (resp., *ρ*) on *S* is a *left translation* (resp., *right translation*) of *S* if *λ*(*xy*) = (*λx*)*y* (resp., (*xy*)*ρ* = *x*(*yρ*)) for all *x*, *y* ∈ *S*. A left translation *λ* and a right translation *ρ* are *linked* if *x*(*λy*) = (*xρ*)*y* in which case the pair (*λ*, *ρ*) is a *bitranslation* of *S*. Denote the set of left translations and that of right translations on *S* by Λ(*S*) and *P*(*S*), respectively. Clearly, Λ(*S*) forms a monoid under the usual composition of functions: (*λλ*′)*x* = *λ*(*λ*′*x*) for all *x* ∈ *S*. Dually, *P*(*S*) forms a monoid under the usual composition of functions: *x*(*ρρ*′) = (*xρ*)*ρ*′ for all *x* ∈ *S*. The set of all bitranslations of *S* forms a submonoid of the direct product Λ(*S*) × *P*(*S*), which is called *the translation hull* of *S*, to be denoted by *Ω*(*S*).

Let *s* be an element of a semigroup *S*. Then the function *λ*_*s*_ defined by *λ*_*s*_ *x* = *sx* for all *x* ∈ *S* is *the inner left translation induced by s*. Dually, we have *inner right translation ρ*_*s*_ *induced by s*. Finally, the pair *π*_*s*_ = (*λ*_*s*_, *ρ*_*s*_) is *the inner bitranslation induced by s*. The set Π(*S*) of all inner bitranslations is *the inner part of Ω*(*S*). From Corollary III.1.7 in Petrich [7], Π(*S*) is an ideal of *Ω*(*S*).

In the sequel, the set of all transformations on a set *Q* written and composed as right (resp., left) operators is denoted by *𝒯* ^*r*^(*Q*) (resp., *𝒯* ^*l*^(*Q*)). The identity mapping on a set *Q* is denoted by *ι*_*Q*_. If *q* ∈ *Q*, then 〈*q*〉 denotes the constant function on *Q* whose value is *q*. Clearly, *𝒯* ^*l*^(*Q*) and *𝒯* ^*r*^(*Q*) are semigroups with their own compositions and *𝒯*_0_^*l*^(*Q*) = {〈*q*〉∣*q* ∈ *Q*} (as left operators) and *𝒯*_0_^*r*^(*Q*) = {〈*q*〉∣*q* ∈ *Q*} (as right operators) are subsemigroups of *𝒯* ^*l*^(*Q*) and *𝒯* ^*r*^(*Q*), respectively.

On the translation hull of a rectangular band, we have the following results which can be found in Section III.7 in Petrich and Reilly [6].

**Lemma 3 (see [6])**

Let *I* × Λ be a rectangular band.

1. *Ω*(*I* × Λ)≅*𝒯* ^*l*^(*I*) × *𝒯* ^*r*^(Λ).
2. *I* × Λ≅Π(*I* × Λ)≅*𝒯*_0_^*l*^(*I*) × *𝒯*_0_^*r*^(Λ).
3. *𝒯*_0_^*l*^(*I*) × *𝒯*_0_^*r*^(Λ) is the kernel of *𝒯* ^*l*^(*I*) × *𝒯* ^*r*^(Λ).

Let *S* be a semigroup and *J* an ideal of *S*. Then *S* is called an *extension* of *J*. From Definition III.5.4 in Petrich [7], an extension *S* of *J* is called *dense* if for each congruence *ρ* on *S*, the fact that the restriction of *ρ* to *J* is the equality relation on *J* implies that *ρ* is the equality relation on *S*.

On dense extensions of rectangular bands, the following results can be obtained as a special case from Theorem III.1.12 and Corollary III.5.5 in [7] and can also be proved easily.

**Lemma 4**

If *S* is a dense extension of a rectangular band *J*, then the following semigroup homomorphism

(1) $$\begin{array}{c} \tau :S\to \Omega \left(J\right),\;s\mapsto s\tau =\left(\lambda^{s},\rho^{s}\right) \end{array}$$

is injective, where for each *s* ∈ *S*,

(2) $$\begin{array}{c} \lambda^{s}:J\to J,\;j\mapsto sj,\;\;\rho^{s}:J\to J,\;j\mapsto js. \end{array}$$

Clearly in this case, *S* is isomorphic to *Sτ* which contains Π(*J*) as an ideal.

Let *S* be a semigroup and let *S* ^1^ be the semigroup obtained from *S* by adjoining an identity if necessary. The syntactic congruence *P*_*L*_ determined by a subset *L* of *S* is the following relation on *S*: {(*x*, *y*) ∈ *S* × *S* | *uxv* ∈ *L* if and only if *uyv* ∈ *L* for all *u*, *v* ∈ *S* ^1^}. In particular, if *L* = {*x*}, we call  *P*_{*x*}_ *the syntactic congruence determined by x* and denote it by *P*_*x*_. Moreover, *x* is called *disjunctive* in *S* if *P*_*x*_ is the equality relation on *S*. It is easy to see that the relation  *P*_*L*_ *saturates* *L* for every subset *L* of *S*; that is, *L* is a union of some *P*_*L*_-classes for every subset *L* of *S*.

Let *A* be an alphabet, let A* be the free monoid generated by *A*, and let 1 be the identity of A*. For any *L*⊆A*, the quotient monoid A*/*P*_*L*_ is called *the syntactic monoid of L*, to be denoted by Syn(*L*). A nonempty set *C* of *A* ^+^ = A*∖{1} is called a *code* over *A* if the fact that

(3) x1x2⋯xm=y1y2⋯yn, xi, yj∈C,  i=1,2,…,m,j=1,2,…,n,

implies that *m* = *n* and *x*_*i*_ = *y*_*i*_, *i* = 1,2,…, *n*.

A submonoid *S* of a monoid *M* is called *stable* in *M* if the fact that *u*, *wu*, *uw* ∈ *S* implies that *w* ∈ *S* for all *u*, *v*, *w* ∈ *M*. It is well known that the monoid C* generated by a code *C* over *A* is stable in A* (see Proposition 2.2.5 in [1]).

A code *C* over *A* is called *complete* if, for any *w* ∈ A*, there exist *u*, *v* ∈ A* such that *uw* *v* ∈ C*, where C* is the monoid generated by *C*. Recall that a complete code *C* over *A* is said to be *synchronized* if xA*∩A*y⊆C* for some *x*, *y* ∈ C* (see details in Proposition 10.1.14 of [1]). On complete synchronized codes, Liu [3] obtained the following algebraic characterizations recently.

**Lemma 5 (see [3])**

A complete code *C* over *A* is synchronized if and only if the kernel of Syn(C*) is a rectangular band.

## 3. A Characterization of Complete Synchronized Codes

This section gives a characterization of complete synchronized codes by using the syntactic monoid Syn(*C* ^+^) of *C* ^+^, the semigroup generated by a code *C* over *A*. To this aim, we need several lemmas.

**Lemma 6**

Let *C* be a code over *A*.

1. For any *x*, *y* ∈ *A* ^+^, *xP*_*C*^+^_ *y* if and only if *xP*_C*_ *y*.
2. The *P*_*C*^+^_-class containing 1 is {1}.
3. Syn(*C* ^+^)∖{1*P*_*C*^+^_} is a subsemigroup of Syn(*C* ^+^).

**Proof**

(1) This follows from the fact that *uxv* ∈ *C* ^+^ if and only if *uxv* ∈ C* for any *x* ∈ *A* ^+^ and *u*, *v* ∈ A*.

(2) Let *w* ∈ A* and *wP*_*C*^+^_1. Since 1 ∉ *C* ^+^ and *C* ^+^ is a union of some *P*_*C*^+^_-classes, it follows that *w* ∉ *C* ^+^. On the other hand, for any *c* ∈ *C*, we have *c* *wP*_*C*^+^_ *c* and *wcP*_*C*^+^_ *c*, whence *cw*, *wc*, *c* ∈ *C* ^+^ by the fact that *C* ^+^ is a union of some *P*_*C*^+^_-classes. Since *C* is a code over *A*, C* is stable. Therefore, *w* ∈ C*. This implies that *w* = 1. Thus, the *P*_*C*^+^_-class containing 1 is {1}.

(3) This follows from (2).

**Lemma 7**

Let *C* be a code over *A*. If the *P*_C*_-class containing 1 is {1}, then Syn(C*)≅Syn(*C* ^+^). Otherwise, Syn(C*)≅Syn(*C* ^+^)∖{1*P*_*C*^+^_}.

**Proof**

If the *P*_C*_-class containing 1 is {1}, by items (1) and (2) in Lemma 6, *P*_*C*^+^_ = *P*_C*_ in this case, and so Syn(C*) is isomorphic to Syn(*C* ^+^).

If the *P*_C*_-class containing 1 is not {1}, then there exists *α* ∈ *A* ^+^ such that 1*P*_C*_ *α*. Moreover, Syn(*C* ^+^)∖{1*P*_*C*^+^_} is a subsemigroup of Syn(*C* ^+^) by item (3) of Lemma 6. In the sequel, we show that the following

(4) $$\begin{array}{c} \psi :\text{Syn}\left(C^{*}\right)\to \text{Syn}\left(C^{+}\right)\setminus \left\{1P_{C^{+}}\right\},\\ \left(xP_{C^{*}}\right)\psi =\{\begin{array}{cc} \alpha P_{C^{+}}, & \text{if}\;\;x=1,\\ xP_{C^{+}}, & \text{if}\;\;x\neq 1, \end{array} \end{array}$$

is a semigroup isomorphism. We first show that *ψ* is well defined. In fact, let *x*, *y* ∈ A* and *xP*_C*_ = *yP*_C*_. We divide the discussion into the following four cases.

- *x* = *y* = 1. In this case, (*xP*_C*_)*ψ* = (*yP*_C*_)*ψ* = *αP*_*C*^+^_.
- *x* = 1, *y* ≠ 1. In this case, (*xP*_C*_)*ψ* = *αP*_*C*^+^_ and (*yP*_C*_)*ψ* = *yP*_*C*^+^_. Observe that *αP*_C*_1 = *xP*_C*_ *y*; it follows that *αP*_C*_ *y* whence *αP*_*C*^+^_ *y* from item (1) in Lemma 6. This implies that (*xP*_C*_)*ψ* = (*yP*_C*_)*ψ*.
- *x* ≠ 1, *y* = 1. This is the dual of case (ii).
- *x* ≠ 1, *y* ≠ 1. This follows from item (1) in Lemma 6.

By similar methods, we can show that *ψ* is injective, and the surjectivity of *ψ* is obvious.

On the other hand, for any *xP*_C*_, *yP*_C*_ ∈ Syn(C*), we assert that

(5) $$\begin{array}{c} \left[\left(xP_{C^{*}}\right)\left(yP_{C^{*}}\right)\right]\psi =\left(xP_{C^{*}}\right)\psi \left(yP_{C^{*}}\right)\psi \end{array}$$

and so *ψ* is a semigroup morphism. In fact, we have the following cases.

(a) *x* = *y* = 1. In this case,
  (6) $$\begin{array}{c} \left[\left(xP_{C^{*}}\right)\left(yP_{C^{*}}\right)\right]\psi =\left(1P_{C*}\right)\psi =\alpha P_{C^{+}},\\ \left(xP_{C^{*}}\right)\psi \left(yP_{C^{*}}\right)\psi =\left(\alpha P_{C^{+}}\right)\left(\alpha P_{C^{+}}\right)=\alpha^{2}P_{C^{+}}. \end{array}$$
  Observe that 1*P*_C*_ *α*, *α* ^2^ *P*_C*_ *α*. This implies that *α* ^2^ *P*_*C*^+^_ *α* by item (1) in Lemma 6 whence [(*xP*_C*_)(*yP*_C*_)]*ψ* = (*xP*_C*_)*ψ*(*yP*_C*_)*ψ*.
(b) *x* = 1, *y* ≠ 1. In this case,
  (7) $$\begin{array}{c} \left[\left(xP_{C^{*}}\right)\left(yP_{C^{*}}\right)\right]\psi =\left(yP_{C^{*}}\right)\psi =yP_{C^{+}},\\ \left(xP_{C^{*}}\right)\psi \left(yP_{C^{*}}\right)\psi =\left(\alpha P_{C^{+}}\right)\left(yP_{C^{+}}\right)=\left(\alpha y\right)P_{C^{+}}. \end{array}$$
  Observe that 1*P*_C*_ *α*, *yP*_C*_ *αy*. This implies that *yP*_*C*^+^_ *αy* by item (1) in Lemma 6 again whence [(*xP*_C*_)(*yP*_C*_)]*ψ* = (*xP*_C*_)*ψ*(*yP*_C*_)*ψ*.
(c) *x* ≠ 1, *y* = 1. This is the dual of case (b).
(d) *x* ≠ 1, *y* ≠ 1. This is obvious.

**Lemma 8**

If *M* is a monoid with identity 1 and *M*∖{1} is a subsemigroup of *M*, then *M* has a kernel if and only if *M*∖{1} has a kernel. If this is the case, the two kernels are equal.

**Proof**

Observe that

(8) {N⊆M ∣ N  is  an  ideal  of  M}  ={N⊆M∖{1} ∣ N  is  an  ideal  of  M∖{1}}∪{M};

the result follows.

Combining Lemmas 5, 6, 7, and 8, we have the following result.

**Theorem 9**

A complete code *C* over *A* is a synchronized code if and only if the kernel of Syn(*C* ^+^) is a rectangular band.

## 4. Main Results

Let *I* and Λ be two nonempty sets and *∅* ≠ *K*⊆*𝒯* ^*l*^(*I*) × *𝒯* ^*r*^(Λ). Assume that *p*_1_ and *p*_2_ are the projections onto the first and second components of *𝒯* ^*l*^(*I*) × *𝒯* ^*r*^(Λ), respectively. For (*i*, *λ*), (*i*_0_, *λ*_0_) ∈ *I* × Λ, we denote

(9) $$\begin{array}{c} \Omega_{\left(i_{0},\lambda_{0}\right)}^{\left(i,\lambda \right)}=\left\{\left(F,\Phi \right)\in p_{1}\left(K\right)\times p_{2}\left(K\right)\;|\;\left(Fi,\lambda \Phi \right)=\left(i_{0},\lambda_{0}\right)\right\}. \end{array}$$

**Theorem 10**

Let *C* be a complete synchronized code over an alphabet *A* such that *C* ≠ *A* and *C* ^+^ is a *P*_*C*^+^_-class. Then Syn(*C* ^+^) is isomorphic to a submonoid *K* of *𝒯* ^*l*^(*I*) × *𝒯* ^*r*^(Λ) for some nonempty sets *I* and Λ with (|*I* | , |Λ|)≠(1,1), and the following conditions hold:

1. *𝒯*_0_^*r*^(*I*) × *𝒯*_0_^*r*^(Λ)⊆*K*,
2. *K*∖{(*ι*_*I*_, *ι*_Λ_)} is a subsemigroup of *K*,
3. there exists (*i*_0_, *λ*_0_) ∈ *I* × Λ such that the identity *Ω*_(*i*_0_,*λ*_0_)_^(*i*,*λ*)^ = *Ω*_(*i*_0_,*λ*_0_)_^(*j*,*μ*)^ implies that (*i*, *λ*) = (*j*, *μ*) for all (*i*, *λ*), (*j*, *μ*) ∈ *I* × Λ and the identity (*Fi*_0_, *λ*_0_Φ) = (*i*_0_, *λ*_0_) implies that (*F*, Φ)∈{(*ι*_*I*_, *ι*_Λ_), (〈*i*_0_〉, 〈*λ*_0_〉)} for all (*F*, Φ) ∈ *K*.

The above submonoid *K* will be called (*i*_0_, *λ*_0_)-submonoid of *𝒯* ^*l*^(*I*) × *𝒯* ^*r*^(Λ).

**Proof**

Let *M* = Syn(*C* ^+^) and denote the set of *P*_*C*^+^_-classes with representatives from *T* by $\bar{T}$ for *T*⊆A*. Since *C* ^+^ is a *P*_*C*^+^_-class, we can also let $e=\bar{C^{+}}$. Obviously, *e* is an idempotent in *M*.

We first assert that $\left\{e,\bar{1}\right\}$ is stable and *e* is disjunctive in *M*. Let *w* ∈ A* and *c* ∈ *C*. Then $e=\overset{-}{c}$. If $\bar{w}\bar{1},\bar{1}\bar{w}\in \{e,\bar{1}\}$, then $\bar{w}\in \{e,\bar{1}\}$ obviously. Moreover, let $\bar{w}e,e\bar{w}\in \{e,\bar{1}\}$. Since the *P*_*C*^+^_-class containing 1 is {1} by Lemma 6, we have $e=\bar{w}e=e\bar{w}$. This implies that $\bar{c}=\bar{wc}=\bar{cw}$. Since *C* ^+^ is a single *P*_*C*^+^_-class, we have *wc*, *cw*, *c* ∈ *C* ^+^. Because C* is stable, it follows that *w* ∈ C*. This implies that $\bar{w}\in \{e,\bar{1}\}$. On the other hand, let *p*, *q* ∈ A* and $\bar{p}P_{e}\bar{q}$ in *M*. Then for all *s*, *t* ∈ A*, $\bar{spt}=e=\bar{C^{+}}$ if and only if $\bar{sqt}=e=\bar{C^{+}}$. Since *C* ^+^ is a single *P*_*C*^+^_-class, it follows that *spt* ∈ *C* ^+^ if and only if *sqt* ∈ *C* ^+^, and so *pP*_*C*^+^_ *q*. Thus $\bar{p}=\bar{q}$.

Now, let *J* = *MeM*. We assert that *J* is the kernel of *M*. In fact, *J* is an ideal of *M* clearly. Moreover, since *C* is complete, there exist *u*, *v* ∈ A* such that *uw* *v* ∈ *C* ^+^ for all *w* ∈ A*. Therefore, there exist *m*, *n* ∈ *M* such that *mxn* = *e* for all *x* ∈ *M*. Now, let *I* be an ideal of *M* and *x* ∈ *I*. Then *uxv* = *e* for some *u*, *v* ∈ *M* whence *J* = *MeM* = *M* *uxv* *M*⊆*I*. Thus, *J* is the least ideal of *M* and so is the kernel of *M*. By Theorem 9, *J* is a rectangular band.

If *J* = {*e*}, then we have *em* = *me* = *e* for any *m* ∈ *M*. This implies that $\overset{-}{c}\;\overset{-}{w}=\overset{-}{w}\;\;\overset{-}{c}=\overset{-}{c}$ for all *c* ∈ *C* and *w* ∈ A*. Since *C* ^+^ is a single *P*_*C*^+^_-class, it follows that *wc*, *cw*, *c* ∈ *C* ^+^. Because C* is stable, we have *w* ∈ C*. Therefore, C* = A* and hence *C* = *A*. A contradiction. Thus, *J* ≠ {*e*}. Now let *ρ* be a congruence on *M* whose restriction to *J* is the identity relation on *J*. Assume that *x* ∈ *M* and *xρ* *e*. Then *xeρeρex*, where *e*, *xe*, *ex* ∈ *J* and thus *e* = *xe* = *ex*. Since $\left\{e,\bar{1}\right\}$ is stable in *M*, it follows that $x\in \{e,\bar{1}\}$. If $x=\bar{1}$, then $\bar{1}\rho e$ and for any *y* ∈ *J*, we obtain *yρ* *ye*, *yρ* *ey*, and *ye*, *y*, *ey* ∈ *J*, whence *y* = *ye* = *ey*. Thus, *J* is a rectangular band with the identity *e*. And hence, *J* = {*e*} by Lemma 1. A contradiction. It follows that *x* = *e* and {*e*} is a *ρ*-class in *M*. Now, let *x*, *y* ∈ *M* and *xρ* *y*. Then for any *u*, *v* ∈ *M*, we have *uxv* *ρ* *uyv*. Observe that {*e*} is a *ρ*-class, it follows that *uxv* = *e* if and only if *uyv* = *e*. Therefore, *xP*_*e*_ *y* in *M*. By the disjunctiveness of *e* in *M*, we have *x* = *y*. We conclude that *ρ* is the equality relation on *M*. Thus, *M* is a dense extension of *J*.

By Lemma 4, *M* is isomorphic to a subsemigroup of *Ω*(*J*) containing the inner part Π(*J*) as an ideal. Since *J* is a rectangular band, *J* is isomorphic to the product *I* × Λ of a left zero semigroup *I* and a right zero semigroup Λ. Observe that *J* ≠ {*e*}, |*J* | = |*I* × Λ | >1, whence (|*I* | , |Λ|)≠(1,1). By Lemma 3, *Ω*(*I* × Λ)≅*𝒯* ^*l*^(*I*) × *𝒯* ^*r*^(Λ), and in this isomorphism Π(*I* × Λ)≅*𝒯*_0_^*l*^(*I*) × *𝒯*_0_^*r*^(Λ). Therefore, *M* is isomorphic to a subsemigroup *K* of *𝒯* ^*l*^(*I*) × *𝒯* ^*r*^(Λ) and *K* contains *𝒯*_0_^*l*^(*I*) × *𝒯*_0_^*r*^(Λ) as an ideal. Furthermore, since *M* is a monoid, *K* has an identity. Let (*F*, Φ) be the identity of *K*. Then for any (〈*i*〉, 〈*λ*〉) ∈ *𝒯*_0_^*l*^(*I*) × *𝒯*_0_^*r*^(Λ),

(10) $$\begin{array}{c} \left(F,\Phi \right)\left(\langle i\rangle ,\langle \lambda \rangle \right)=\left(\langle i\rangle ,\langle \lambda \rangle \right)\left(F,\Phi \right)=\left(\langle i\rangle ,\langle \lambda \rangle \right), \end{array}$$

whence (*Fi*, *λ*Φ) = (*i*, *λ*). This implies that (*F*, Φ) = (*ι*_*I*_, *ι*_Λ_). Thus, *K* is a submonoid of *𝒯* ^*l*^(*I*) × *𝒯* ^*r*^(Λ). Since *M*∖{1} is a subsemigroup of *M*, it follows that *K*∖{(*ι*_*I*_, *ι*_Λ_)} is a subsemigroup of *K*. Thus, Conditions (1) and (2) hold.

Since *J* is the kernel of *M* and *e* is an idempotent in *J*, it follows that the image of *e* in *K* is the form *a* = (〈*i*_0_〉, 〈*λ*_0_〉) for some *i*_0_ ∈ *I* and *λ*_0_ ∈ Λ. Since *e* is disjunctive in *M*, *a* is disjunctive in *K*. Let (*i*, *λ*), (*j*, *μ*) ∈ *I* × Λ and *Ω*_(*i*_0_,*λ*_0_)_^(*i*,*λ*)^ = *Ω*_(*i*_0_,*λ*_0_)_^(*j*,*μ*)^. Then,

(11) $$\begin{array}{c} \left(Fi,\lambda \Phi \right)=\left(i_{0},\lambda_{0}\right)\;\text{iff}\;\;\left(Fj,\mu \Phi \right)=\left(i_{0},\lambda_{0}\right) \end{array}$$

for any (*F*, Φ) ∈ *p*_1_(*M*) × *p*_2_(*M*). This implies that

(12) $$\begin{array}{c} \left(\langle Fi\rangle ,\langle \lambda \Phi \rangle \right)=a\;\text{iff}\;\;\left(\langle Fj\rangle ,\langle \mu \Phi \rangle \right)=a, \end{array}$$

for any (*F*, Φ) ∈ *p*_1_(*M*) × *p*_2_(*M*). Thus,

(13) $$\begin{array}{c} \left(F,\Psi \right)\left(\langle i\rangle ,\langle \lambda \rangle \right)\left(G,\Phi \right)=a\;\text{iff}\;\;\left(F,\Psi \right)\left(\langle j\rangle ,\langle \mu \rangle \right)\left(G,\Phi \right)=a, \end{array}$$

for any (*F*, Ψ), (*G*, Φ) ∈ *K*. This shows that (〈*i*〉, 〈*λ*〉)*P*_*a*_(〈*j*〉, 〈*μ*〉) and so (〈*i*〉, 〈*λ*〉) = (〈*j*〉, 〈*μ*〉) since *a* is disjunctive in *K*. Thus (*i*, *λ*) = (*j*, *μ*).

Finally, let (*F*, Φ) ∈ *K* and (*Fi*_0_, *λ*_0_Φ) = (*i*_0_, *λ*_0_). Then,

(14) $$\begin{array}{c} \left(F,\Phi \right)\left(\langle i_{0}\rangle ,\langle \lambda_{0}\rangle \right)=\left(\langle Fi_{0}\rangle ,\langle \lambda_{0}\rangle \right)=\left(\langle i_{0}\rangle ,\langle \lambda_{0}\rangle \right). \end{array}$$

Dually, we have (〈*i*_0_〉, 〈*λ*_0_〉)(*F*, Φ) = (〈*i*_0_〉, 〈*λ*_0_〉). Since *e* is stable in *M*, it follows that *a* is stable in *K*. Hence, (*F*, Φ)∈{(〈*i*_0_〉, 〈*λ*_0_〉), (*ι*_*I*_, *ι*_Λ_)}. Therefore, Condition (3) is also satisfied.

We end our paper by giving an example to illustrate our result.

**Example 11**

Let *A* = {*a*, *b*} and *C* = b*a ^+^ *b*. Then *C* ^+^ = A*ab. It is routine to check that *C* is a synchronized code and *C* ^+^ is a single *P*_*L*_-class. Moreover, we can also check that $\text{Syn}(C^{+})=\{\bar{A^{*}ab},\bar{A^{*}bb},\bar{A^{*}a},\bar{b},\bar{1}\}$.

On the other hand, let *I* = {1} and Λ = {1,2, 3}. Denote

(15) $$\begin{array}{c} M_{1}=\left\{\left(\langle 1\rangle ,\langle 1\rangle \right),\left(\langle 1\rangle ,\langle 2\rangle \right),\left(\langle 1\rangle ,\langle 3\rangle \right),\left(\langle 1\rangle ,\alpha \right),\left(\iota_{I},\iota_{\Lambda }\right)\right\}, \end{array}$$

where

(16) $$\begin{array}{c} \alpha :\Lambda \to \Lambda ,1\mapsto 2,2\mapsto 2,3\mapsto 1. \end{array}$$

Then *M*_1_ is a (1,1)-submonoid of *𝒯* ^*l*^(*I*) × *𝒯* ^*r*^(Λ). In fact, observe that *α* ^2^ = 〈2〉, *M*_1_ is submonoid of *𝒯* ^*l*^(*I*) × *𝒯* ^*r*^(Λ), and *M*_1_∖{(*ι*_*I*_, *ι*_Λ_)} is a subsemigroup of *M*_1_. Let *λ*, *μ* ∈ Λ. If *λ* = 1, *μ* = 2 or *λ* = 1, *μ* = 3, then there exists Φ = *ι*_Λ_ ∈ *p*_2_(*M*_1_) such that *λ*Φ = 1, *μ*Φ = *μ*. If *λ* = 2, *μ* = 3, then there exists Φ = *α* ∈ *p*_2_(*M*_1_) such that *λ*Φ = 2, *μ*Φ = 1. Furthermore, if (*F*, Φ) ∈ *M*_1_ with *F*1 = 1, 1Φ = 1, then *F* = 〈1〉 = *ι*_*I*_ and Φ = 〈1〉 or Φ = *ι*_Λ_. Thus, *M*_1_ is a (1,1)-submonoid of *𝒯* ^*l*^(*I*) × *𝒯* ^*r*^(Λ). It is easy to see that

(17) ψ:Syn(C+)→M1,  A∗ab¯↦(〈1〉,〈1〉),A∗bb¯↦(〈1〉,〈2〉),  A∗a¯↦(〈1〉,〈3〉),b¯↦(〈1〉,α),  1¯↦(ιI,ιΛ)

is an isomorphism from Syn(*C* ^+^) onto *M*_1_.
